# Probiotic preparations in mitigating chemotherapy-induced oral mucositis: therapeutic efficacy, mechanisms, and clinical translation potential

**DOI:** 10.3389/fcimb.2026.1870000

**Published:** 2026-07-08

**Authors:** Xiaochen Xiang, Yangjinyu Pei, Qiang Wang

**Affiliations:** Wuhan Asia General Hospital, Wuhan University of Science and Technology, Wuhan, Hubei, China

**Keywords:** chemotherapy-induced oral mucositis, immunomodulation, mucosal barrier, oral microbiota, probiotics

## Abstract

Chemotherapy-induced oral mucositis (CIOM) is a prevalent toxic side effect of cancer treatment, severely compromising patients’ quality of life, nutritional intake, and treatment adherence. Its pathogenesis has evolved from the traditional model of simple epithelial damage to a complex pathological process involving the interplay of chemotherapy toxicity, host immunity, and oral microbiota. Research indicates that chemotherapy can disrupt the oral microbiota, promoting the proliferation of pathogenic bacteria and exacerbating damage to the mucosal barrier and local inflammatory responses. Current clinical interventions, such as mouth rinses and cryotherapy, have limited efficacy and lack standardized protocols. In recent years, modulating the oral microbiota has emerged as a promising therapeutic strategy. Probiotic preparations have demonstrated potential in clinical studies to alleviate CIOM severity through mechanisms including competitive colonization, metabolic regulation, and immunomodulation. This review systematically summarizes the clinical manifestations, epidemiological characteristics, pathogenesis, and existing treatment strategies of CIOM. It highlights the critical role of the oral microbiota in CIOM pathogenesis and further outlines the promising application prospects of microbiome-targeted interventions, particularly probiotic preparations, aiming to provide novel insights for CIOM prevention and treatment.

## 1 Introduction

Oral mucositis (OM) is an oral disease mediated by multiple factors including infection, trauma, and immune abnormalities, manifesting as inflammatory or ulcerative lesions on the oral mucosal epithelium (Al-Rudayni et al., 2021). The mucous membranes of the tongue, tongue base, buccal mucosa, and soft palate lack a stratum corneum or exhibit low levels of keratinization, making them susceptible to invasion by pathogenic microorganisms, immune disorders, physical and chemical damage, and the effects of drug therapy (Zhou et al., 2022). Under normal circumstances, oral mucositis typically presents as a self-limiting condition. However, in cancer patients, Chemotherapy-induced oral mucositis (CIOM) induced by cytotoxic therapy manifests with significantly more severe symptoms, affecting treatment outcomes, physiological functions, quality of life, treatment tolerance, and healthcare costs. In some cancer patient populations, the incidence of CIOM can be as high as 90% (Shuai et al., 2019) and is associated with an increased risk of mortality.

The incidence of CIOM varies epidemiologically across different regions, tumor types, and treatment modalities. It is essential to comprehend these epidemiological traits for risk management and CIOM prevention. CIOM typically develops 4–7 days after chemotherapy, with symptoms peaking at 10–14 days (Zhang et al., 2023). Patients undergoing high-dose chemotherapy as pre-treatment for hematopoietic stem cell transplantation have an OM incidence of 70%–100%, with 67.4% experiencing grade ≥3 lesions. For head and neck cancer patients, the OM incidence ranges from 50% to 90%, and 60% of them develop grade ≥3 lesions (Yoshimatsu et al., 2023). Combination chemotherapy regimens carry a higher risk than single-agent therapies. For example, the incidence of grade ≥3 OM in non-Hodgkin lymphoma patients receiving the CHOEP-14 regimen (CHOEP: Cyclophosphamide+Hydroxydaunorubicin+Oncovin+Etoposide+ Prednisone) is 10.4%, while that in breast cancer patients treated with the AC regimen (AC: Adriamycin + Cyclophosphamide) is 13.64%. CIOM is mainly characterized by mucosal erythema, ulceration, and pain; severe cases may involve bleeding, and mucosal damage increases the risk of secondary infections (Basile et al., 2019). The incidence and severity of CIOM exhibit significant heterogeneity, influenced by tumor type, treatment regimen, and patient-specific conditions. In recent years, the epidemiological features of CIOM have also shown new manifestations as treatment and care methods have evolved and improved.

Despite the devastating impact of CIOM on tumor treatment, the efficacy of current clinical interventions (including nutritional support, mucosal protectants, cryotherapy, and antibiotics) remains limited (Tanideh et al., 2019). Moreover, medical centers lack unified standards for CIOM treatment protocols, and existing therapeutic approaches struggle to accommodate all tumor types or treatment modalities. There is an urgent need to identify safer and more effective therapeutic strategies.

The complex pathogenesis of CIOM has been extensively studied and demonstrated. New evidence suggests that the pathogenesis of chemotherapy-induced oral mucositis (CIOM) is not driven solely by chemotherapy toxicity, but rather by a complex interplay between host immunity, chemotherapy-induced damage, and the oral microbiome (Li et al., 2020; Bruno et al., 2022; Medeiros et al., 2023). Disruption of the oral microbiota impairs the mucosal barrier, facilitates bacterial invasion, and accelerates the progression of mucositis. This suggests that modulating the oral microbiota may represent a potential therapeutic intervention for CIOM. A systematic review and meta-analysis of randomized controlled trials have demonstrated that adjunctive therapy with probiotics can safely and effectively reduce the incidence of OM (Yang et al., 2024). However, the protective mechanisms of probiotics remain unclear. Several animal studies have preliminarily explored that probiotics could regulate epithelial function, maintain mucosal barrier integrity, enhance host immunity, and inhibit the colonization of pathogenic bacteria (Ukena et al., 2007; Li et al., 2025; Souza et al., 2026).

This review aims to present the latest advances in the clinical manifestations, epidemiology, pathogenesis, and existing or emerging intervention strategies of CIOM, thereby providing novel insights for CIOM research and therapeutic practice.

## 2 Epidemiological characteristics and pathogenesis of CIOM

In the early 20th century, the German chemist Paul Ehrlich proposed the concept of “treating diseases with chemical drugs,” marking the dawn of the cancer chemotherapy era (Ehrlich, 1909). Over a century of development, chemotherapy has evolved from a palliative treatment for advanced cancers to a core component of comprehensive cancer therapy, significantly altering the public’s pessimistic perception of oncological drug treatments. Currently, the focus of tumor research and clinical practice has shifted toward “precision medicine,” encompassing the detection of key driver gene mutations, identification of novel therapeutic targets, and development of targeted drugs and immune checkpoint inhibitors (Yan et al., 2021). Concurrently, with the advancement of therapeutic innovations, the spectrum and severity of treatment-related adverse reactions have been continuously evolving — novel targeted therapies and immunotherapies deliver improved therapeutic efficacy, yet are concurrently accompanied by unique novel toxicity profiles (Kroschinsky et al., 2017).

OM is one of the most common side reactions to chemotherapy, and the heterogeneity of its epidemiological characteristics poses a core challenge in clinical management (Franco et al., 2023). Traditional views regarded OM as a purely epithelial cell injury process: chemotherapeutic drugs directly damage to the basal layer cells of the mucosa by inhibiting DNA synthesis and cell division in epithelial stem cells, leading to the loss of cell renewal capacity, subsequent clonal cell death, tissue atrophy, and ulcer formation (Lockhart and Sonis, 1981; Tavhare, 2024). This “single-factor epithelial cell model” long dominated CIOM mechanism research but failed to explain the clinical phenomenon that “patients receiving the same chemotherapy regimen exhibit significant variations in OM severity.” With advances in clinical biological research, a “multifactorial interaction model” for CIOM has emerged: studies have confirmed that CIOM development results from the combined effects of chemotherapy toxicity, host immunity, and the mucosal microenvironment, with inflammatory factors produced by mucosal cells and immune cells in the submucosal layer playing a key regulatory role (Hong et al., 2019). For instance, elevated levels of tumor necrosis factor-α (TNF-α), interleukin-1 (IL-1), and interleukin-6 (IL-6) in peripheral blood are significant positive correlation with the severity of OM following chemotherapy (Hall et al., 1995). Animal model studies have demonstrated that the gene expression levels of interleukin-1β (IL-1β) and TNF-α in mucosal tissues are significantly associated with the progression of mucositis (Sonis et al., 2000). Collectively, these findings indicate that the pathological process of CIOM involves a cascade reaction among oral mucosal cells, tissues, and the extracellular matrix, which can be divided into five sequential stages:

1. Initiation stage: Chemotherapy drugs induce DNA damage in epithelial cells, activating oxidative stress responses.
2. Pro-inflammatory mediator upregulation stage: Damaged cells release pro-inflammatory cytokines (e.g., TNF-α, IL-1).
3. Signal transmission and amplification stage: Cytokines diffuse via autocrine/paracrine pathways, activating additional immune cells and epithelial cells.
4. Ulceration with inflammation stage: The integrity of the mucosal epithelium is disrupted, and microbial invasion cause localized inflammation.
5. Healing stage: Inflammation subsides, and epithelial stem cells proliferate to repair the mucosa (Naidu et al., 2004).

**Figure 1:**
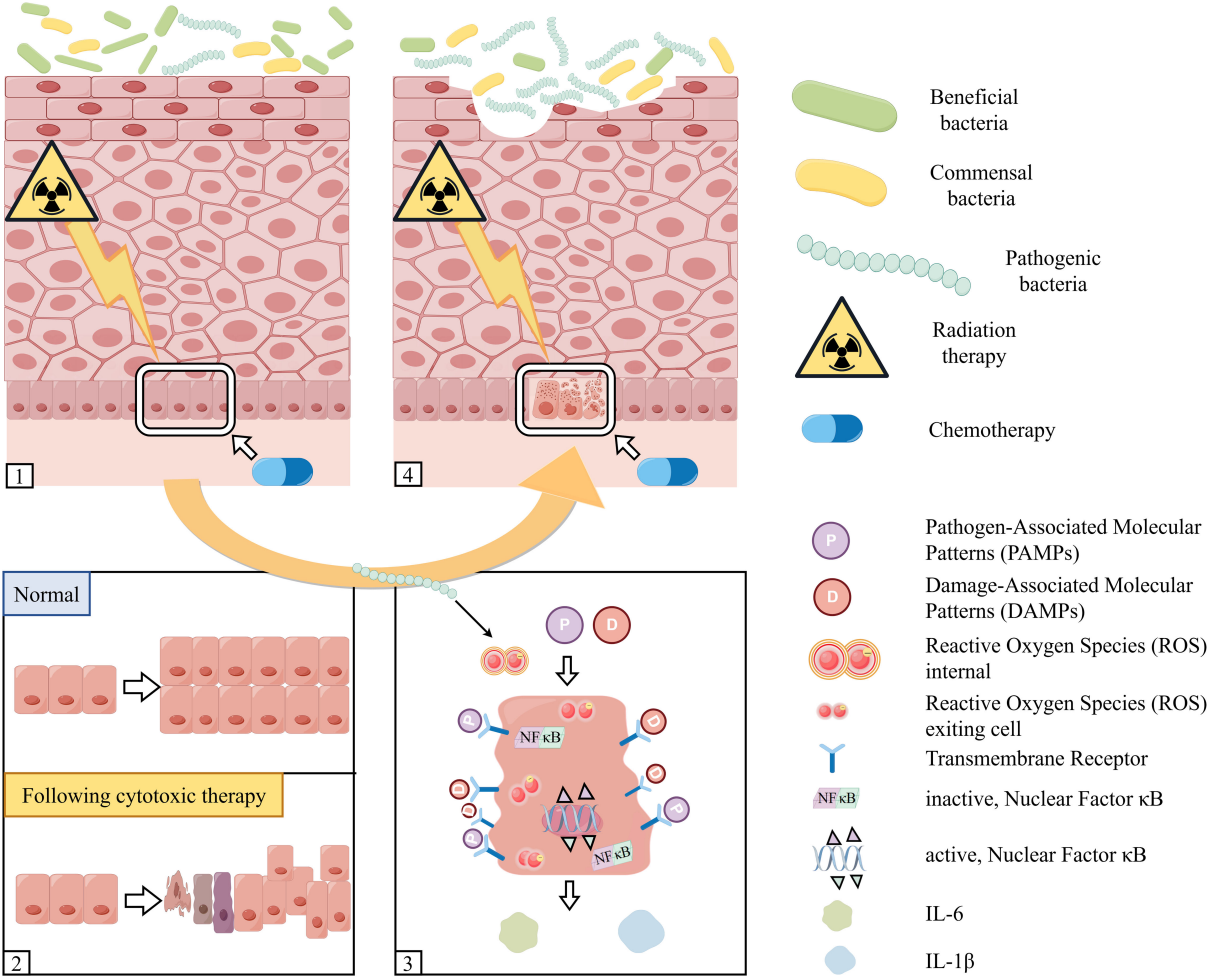
Mechanisms underlying cytotoxic therapy-induced oral mucositis (CIOM). Cytotoxic therapy directly damages mucosal cells, slowing the proliferation and differentiation of basal layer cells or inducing cell death, and releasing endogenous damage-associated molecular patterns (DAMPs). Concurrently, mucosal barrier disruption and neutropenia lead to oral microbiota dysbiosis and pathogenic bacterial overgrowth. Pathogens release exogenous pathogen-associated molecular patterns (PAMPs). DAMPs and PAMPs bind to pattern recognition receptors on the cell surface (e.g., Toll-like receptors) to activate downstream signaling pathways. This process is accompanied by massive production of reactive oxygen species (ROS) both intracellularly and extracellularly, which further exacerbates tissue injury. Activated NF-κB initiates the transcription and expression of pro-inflammatory cytokine genes (e.g., IL-1β, IL-6). These inflammatory cytokines amplify local tissue damage through a positive feedback loop, ultimately leading to typical pathological manifestations including mucosal erythema, erosion, and ulceration.

In recent years, the relationship between the human microbiome and disease has emerged as a cutting-edge focus in medical biology research, with the role of the oral microbiome in the pathogenesis of CIOM gradually gaining attention (Napeñas et al., 2007; Gupta et al., 2019). Germ-free mouse experiments conducted by N Gupta et al. provided direct evidence for the “involvement of oral microbiota in CIOM regulation”: 5-fluorouracil (5-FU)-treated germ-free mice exhibited significantly milder oral epithelial damage compared to SPF-grade mice, accompanied by higher expression of the cell proliferation marker Ki-67 and lower levels of metalloproteinases and inflammatory cytokines (e.g., Matrix Metalloproteinase-9 (MMP-9), IL-6) (Gupta et al., 2019). These findings suggest that oral commensal microbiota may exacerbate CIOM damage by promoting inflammatory responses. Joel J Napeñas et al. conducted a meta-analysis systematically reviewing 13 prospective clinical trials to assess the association between oral microbiota changes and OM. Although the pooled analysis did not reach statistical significance, subgroup analysis revealed that patients with increased abundance of oral pathogens (e.g., *Porphyromonas gingivalis*, *Capnocytophaga*) after chemotherapy had more severe OM (Napeñas et al., 2007). This study suggests a potential causal relationship between cancer chemotherapy, oral microbiota alterations, and OM, though validation in larger cohort studies is required. Collectively, the above studies demonstrate that cytotoxic therapy-induced oral mucositis is primarily driven by direct chemotherapy-mediated epithelial damage. Meanwhile, pathogenic microorganisms act as crucial biological modulators to exacerbate inflammatory signaling, impair epithelial repair, and increase susceptibility to ulcers and infections. As illustrated in **Figure 1**, gut microbiota dysbiosis serves as a regulatory factor and amplifier rather than an initiator of tissue injury during this pathological process (Obeagu, 2026).

The vast majority of commonly used clinical chemotherapeutic agents carry the potential toxicity to induce oral mucositis, including cyclophosphamide, doxorubicin, vincristine, etoposide, methotrexate, 5-FU, cisplatin, and carboplatin. The risk of OM varies by drug: the incidence of grade 3–4 OM associated with 5-FU monotherapy exceeds 15%, and concurrent radiotherapy may further increase the risk of oral and gastrointestinal mucositis by an additional 30% (Peterson et al., 2015). Patients undergoing hematopoietic stem cell transplantation have a significantly higher OM incidence than general cancer patients due to high-dose chemotherapy toxicity in conditioning regimens, with most studies reporting a grade 3–4 OM incidence of 30%–50% (Choe et al., 2022). Additionally, agents including capecitabine and irinotecan are associated with a high incidence of mucositis (Ribeiro et al., 2016).Addition, while the precision of targeted therapy primarily focuses on tumor cells, its toxic effects on mucosal tissues remain significant and cannot be overlooked, although targeted therapies have markedly improved the survival rates of cancer patients, they have not reduced the risk of mucosa-associated adverse reactions (Elting et al., 2013; Li et al., 2023).

The negative impact of CIOM on tumor treatment outcomes has been confirmed by clinical data. When the incidence of grade 3–4 OM exceeds 5%–15%, approximately 35% of patients experience chemotherapy cycle delays, 60% require chemotherapy dose reduction, and 30% are forced to discontinue the chemotherapy regimen; among grade 3–4 OM patients receiving standard-dose chemotherapy, 70% require nutritional support via nasogastric tubes or gastrostomy tubes (Sonis et al., 2001). These data indicate that OM is one of the chemotherapy-related “dose-limiting toxicities” in cancer treatment, and the quality of its clinical management directly impacts the efficacy of tumor therapy and patients’ quality of life. Currently, multidisciplinary and interdisciplinary researchers have achieved remarkable progress in the pathogenesis, risk prediction and therapeutic strategies of CIOM. However, critical unresolved issues remain, including the causal relationship between the oral microbiota and CIOM and the validation of efficacy for novel interventional strategies.

## 3 Alterations in oral microbiota induced by cancer chemotherapy

The human body harbors multiple microbial ecosystems, and the oral cavity is colonized by a highly diverse community of microorganisms. Within their respective microenvironments, resident microbes maintain a synergistic or symbiotic homeostatic equilibrium through interactions involving nutrients, metabolic byproducts, potential of hydrogen (pH), and temperature (Min et al., 2023). Perturbations to local or systemic factors can disrupt this equilibrium, triggering dysbiosis and the subsequent overproliferation of potential pathogenic bacteria (Bugshan et al., 2017). Chemotherapy plays a pivotal role in tumor control and treatment (Dian et al., 2018), yet its cytotoxicity readily induces microbial dysbiosis, impairs the mucosal barriers of the oral and gastrointestinal tracts, and triggers mucosal inflammation (Lee et al., 2016; Yi et al., 2021). While the cytotoxicity of antineoplastic agents is the primary etiological factor of injury, interactions between oral microbial symbionts and host mucosal tissues may modulate the pathological progression of mucosal damage (Arfken et al., 2019).

To elucidate the effects of chemotherapy on the oral microbial community, Bo-Young Hong’s team conducted a prospective observational study enrolling 49 cancer patients undergoing chemotherapy with 5-FU or doxorubicin. Oral samples were collected prior to, during, and following chemotherapy, and subjected to 16S rRNA gene sequencing and epithelial transcriptome analysis (Hong et al., 2019). Results revealed a significant reduction in oral microbial diversity following chemotherapy, accompanied by increased abundances of pathogenic bacteria (e.g., *Prevotella* spp., *Porphyromonas* spp.). Concurrently, patients with OM exhibited a markedly higher degree of oral dysbiosis compared with those without OM. This study confirms that chemotherapy-induced oral dysbiosis exacerbates epithelial damage and acts as a key regulatory factor in the pathogenesis of CIOM (Hong et al., 2019).

The study by the Napeñas team further supports this conclusion: A 16S rRNA gene clone analysis of oral bacteria in 9 breast cancer patients before and after chemotherapy identified 41 bacterial species across pre- and post-chemotherapy samples, with Staphylococcus haemolyticus and Streptococcus mitis as the main dominant species. Twenty-five species were present (species that appeared exclusively after chemotherapy belong to the family *Lachnospiraccae*, genera *Acidaminococcus*, *Clostridiales*, *Oribacterium*, *Johnsonella*, *Peptostreptococcus*, *Aggregatibacter*, *Haemophilus*, and *Bacteroidetes*, in addition to species such as *Filifactor alocis*, *Veillonella parvula*, *Lactobacillus gasseri*, *Granulicatella adicans*, and *Selenomonas noxia*.) after chemotherapy. Following chemotherapy, the average number of bacterial species per patient increased by 2.6 (standard deviation = 4.7, *P* = 0.052), However, this study only preliminarily explored the potentially complex changes in oral microbiota during chemotherapy. Due to factors such as sample size and control of confounding variables, it is not yet fully clear whether changes in oral bacterial communities can be entirely attributed to the chemotherapy regimen (Napeñas et al., 2010). Fainstein et al. identified 152 pre-chemotherapy colonizing bacterial strains and fungi in 33 adult patients, with Gram-positive bacteria as the predominant group. Sixty-eight percent of these microbial communities were eradicated post-chemotherapy, while 33 novel strains emerged concurrently (Fainstein et al., 1981). Sixou et al. observed more alterations in the oral microbiota during chemotherapy in 16 patients with acute myeloid leukemia (AML) or acute lymphoblastic leukemia (ALL), with fluctuating levels of Streptococcus viridans and Staphylococcus spp. relative to healthy controls (Sixou et al., 1998). The lipopolysaccharides (LPS) produced by such pathogenic bacteria, acting as pathogen-associated molecular patterns (PAMPs), are recognized by pattern recognition receptors on the surface of host immune cells (such as TLR4). This recognition activates intracellular inflammatory signaling pathways, enhancing the activation level and function of pro-inflammatory immune cells, prompting them to secrete large amounts of pro-inflammatory cytokines such as TNF-α, IL-6, and IL-1β. These cytokines enter the bloodstream, spread throughout the body, and ultimately trigger systemic inflammation.

Beyond bacteria, fungi also contribute to post-chemotherapy perturbations in the oral microbiota. A gene expression analysis study demonstrated a marked increase in the abundance of Fusobacterium nucleatum—a conditionally pathogenic fungus—at OM lesion sites during chemotherapy-induced dysbiosis (Hong et al., 2019). Toxic metabolites secreted by Fusobacterium nucleatum upregulate the expression of Tumor Necrosis Factor-α (TNF-α), C-C motif chemokine ligand 20(CCL20), Interleukin-17C (IL-17C), C-X-C motif chemokine ligand 2(CXCL2), phorbol-12-myristate-13-acetate-induced protein 1(PMAIP1), Defensin Beta 4A (DEFB4A), and Defensin Beta 103A (DEFB103A) genes; among these, TNF activates the NF-κB pathway to trigger epithelial cell apoptosis or necroptosis via the extrinsic pathway (Hong et al., 2019). This mechanism further elucidates the molecular underpinnings of oral dysbiosis exacerbating CIOM-associated mucosal damage.

## 4 Association between oral microbiota and oral mucositis

Previous studies have provided substantial evidence that gut bacteria modulate the pathological progression of intestinal mucositis, mucosal permeability, and intestinal epithelial repair by regulating intestinal inflammation (Chaudhry et al., 2023). Given that the oral and intestinal mucosae are components of the same digestive tract, the concept of host-microbiota interactions in intestinal mucositis can be extrapolated to the pathogenesis of OM (Chaudhry et al., 2023). Cytotoxic therapies disrupt the oral microbial homeostasis by damaging non-keratinized mucosal surfaces and reducing neutrophil counts. Impaired mucosal barrier function enables commensal microbes to initiate pathogenic cascades (Donnelly et al., 2003). Eline Vanlancker’s team directly compared the effects of chemotherapeutic agents and the oral microbiota on OM pathogenesis and healing. In a murine model, they assessed the impacts of 5-FU, the oral microbiota, and their combination on OM resolution. The results demonstrated that 5-FU only slightly delayed OM healing, whereas the load and composition of the oral microbiota exerted a more pronounced effect on the healing process. Mice colonized with pathogenic bacteria exhibited a 2–3-day delay in OM resolution. These findings indicate that the oral microbiota serves as a key regulator of CIOM healing (Vanlancker et al., 2018). An observational study by Laheij AM’s team further confirmed the association between the oral microbiota and OM. The team enrolled 50 patients undergoing hematopoietic stem cell transplantation, collected oral samples regularly for microbiota analysis, and found that patients with oral ulcers had a significantly higher abundance of oral pathogenic bacteria (e.g., *Porphyromonas gingivalis*) compared with ulcer-free patients. Furthermore, the severity of oral ulcers was positively correlated with the abundance of pathogenic bacteria (Laheij et al., 2012).

Oral viruses may also be involved in the pathogenesis of OM. Clinical studies by Y-K Chen’s team demonstrated that patients with OM complicated by herpes simplex virus (HSV) infection presented with more severe symptoms and required more frequent and prolonged use of mouth rinses for symptom alleviation, compared with OM patients without HSV infection. This finding indicates that HSV infection may exacerbate inflammatory responses and thereby accelerate the progression of OM (Chen et al., 2011). Additionally, other oral viruses (e.g., Epstein-Barr virus, cytomegalovirus) may also play a role in OM development in immunocompromised patients, though related research remains limited.

From an etiological perspective, Ziyang Min’s team summarized the molecular mechanisms by which oral dysbiosis promotes OM development. This review indicates that chemotherapy, antibiotics, and other drug therapies can induce oral dysbiosis through three pathways: (1) Directly inhibiting or killing the growth of commensal bacteria;(2) Altering the pH value and nutritional status of the oral microenvironment; (3) Suppressing the host’s immune clearance capacity. Dysregulated oral microbiota can promote the development of OM and delay ulcer healing through multiple mechanisms. For instance, lipopolysaccharide (LPS) and peptidoglycan (PTG) produced by pathogenic bacteria can activate Type 1/Type 2 T helper cell (Th1/Th2)-associated immune-inflammatory responses, thereby facilitating the release of pro-inflammatory cytokines. Short-chain fatty acids (SCFAs) generated by the oral microbiota can exacerbate inflammatory responses by regulating the differentiation of regulatory T cells (Tregs) and promoting the activation of Type 17 T helper cell (Th17) cells (Min et al., 2023). Although the association between oral microbiota and OM has been preliminarily established, current research has three major limitations: First, evidence regarding the oral microbiota in chemotherapy-related oral conditions remains scarce, as most studies have focused on the gut microbiota. Second, existing research has predominantly relied on correlation analysis, without validating causal relationships. Third, the mechanisms underlying interactions between the oral microbiota and host immunity remain incompletely elucidated (Min et al., 2023). Future research should prioritize multi-omics studies examining the “microbiota-host” relationship to elucidate the molecular mechanisms by which oral microbiota modulate CIOM.

## 5 Current therapeutic approaches for chemotherapy-induced oral mucositis

Chemotherapy is one of the most common modalities for cancer treatment. However, 20% to 40% of patients receiving conventional chemotherapy and 80% of those undergoing hematopoietic stem cell transplantation are affected by CIOM (Silverman, 2007; Nomura et al., 2012). Effective prevention and management of CIOM constitute a critical clinical need to improve the quality of life of cancer patients and ensure treatment compliance. Current clinical interventions in use include mouth rinse regimens, oropharyngeal cryotherapy, and oral microbiota modulation, each with its inherent limitations (Ito et al., 2023).

Mouth rinse regimens and oropharyngeal cryotherapy are standard preventive strategies for CIOM. Mouth rinses (e.g., normal saline, sodium bicarbonate solution) reduce the incidence of OM by cleansing the oral cavity and inhibiting pathogenic bacterial colonization; oropharyngeal cryotherapy (e.g., ice chip swishing) exerts a protective effect on the oral mucosa by inducing local vasoconstriction via hypothermia to decrease the exposure concentration of chemotherapeutic agents in the oral mucosa, thereby alleviating mucosal damage (Prakash et al., 2020; Bossi et al., 2024). Multiple randomized controlled trials have confirmed that both interventions reduce the incidence and severity of chemoradiotherapy-associated OM: for instance, the use of sodium bicarbonate mouth rinses in patients with head and neck cancer during radiotherapy decreases the incidence of OM by 20%–30% (Bossi et al., 2024). Ice chip swishing in patients receiving high-dose methotrexate chemotherapy reduces the severity of OM by 1–2 grades (Prakash et al., 2020). However, the efficacy of these approaches remains limited. For example, mouth rinses yield only limited relief for severe OM, while oropharyngeal cryotherapy is poorly tolerated due to poor comfort in a subset of patients (Dodd et al., 2022).

Chlorhexidine, a commonly used antibacterial agent in mouth rinses in clinical practice, has controversial efficacy in the intervention of OM. Multiple studies have demonstrated that it exerts no significant effect on the incidence and severity of radiation- or chemotherapy-induced OM: for instance, a randomized controlled trial enrolling 120 patients with head and neck cancer revealed no significant difference in OM severity between chlorhexidine mouth rinse users and the control group (Dodd et al., 1996). A meta-analysis by Andrea M. Stringer further confirmed that chlorhexidine has limited overall efficacy in managing OM in patients undergoing chemotherapy or radiotherapy, and only marginally reduces the incidence of mild OM. This finding may be attributed to the “non-selective antimicrobial activity” of chlorhexidine—while inhibiting pathogenic bacteria, it also disrupts the oral commensal microbiota, thereby impairing the mucosal repair capacity (Cardona et al., 2017). Photobiomodulation therapy (PBMT) has emerged as an investigational therapeutic modality for OM in recent years, which exerts its effects by promoting epithelial cell proliferation and the resolution of inflammation through photobiomodulation (Gouvêa de Lima et al., 2012). However, its efficacy displays tumor type specificity: a phase III randomized study by Aline Gouvêa de Lima’s team in patients with head and neck cancer receiving concurrent chemoradiotherapy demonstrated that PBMT had no significant effect on the remission of severe OM. In contrast, in patients with non-head and neck cancer, PBMT reduced the severity of OM by approximately one grade (Holvoet et al., 2014). This discrepancy may be attributed to the higher radiotherapy doses and more severe mucosal damage observed in patients with head and neck cancer. In summary, current clinical interventions remain inadequate for meeting the clinical needs of CIOM, highlighting the necessity of developing novel interventional strategies with safety, feasibility, cost-effectiveness, and well-demonstrated efficacy.

Advances in human microbiome research have opened up new avenues for the treatment of CIOM, and studies investigating the association between the oral microbiota and OM have been progressively conducted (Omori et al., 2023). A study by Xia C’s team demonstrated that a modified probiotic mixture (containing Lactobacillus, Bifidobacterium, and other strains) significantly ameliorated the severity of OM in patients with nasopharyngeal carcinoma undergoing concurrent chemoradiotherapy: the incidence of OM in the treatment group was 35% lower than that in the control group, with a 20% reduction in the incidence of grade ≥3 OM. This probiotic mixture enhanced immune responses in patients (e.g., increased the ratio of peripheral blood CD4+ T cells), improved the composition of the gut microbiota (e.g., elevated the abundance of Bifidobacterium), and simultaneously suppressed the expression of inflammatory factors (e.g., TNF-α, IL-6). Animal studies confirmed that this probiotic mixture alleviated the severity of 5-FU-induced OM in mice through mechanisms involving the suppression of inflammatory responses, regulation of apoptosis and intestinal permeability, and restoration of gut microbiota homeostasis (Xia et al., 2021). Dayana Gerhard’s team further validated the protective effect of probiotics against CIOM in a 5-FU-induced immunosuppressed rat model. Probiotic supplementation (Lactobacillus species) reduced inflammatory damage in the oral cavity and intestines, decreased the levels of TNF-α and IL-1β in mucosal tissues, and preserved the integrity of intestinal villi (Gerhard et al., 2017). This study suggests that probiotics may modulate mucosal inflammation via the gut-oral axis, providing novel therapeutic insights for the management of CIOM. Collectively, the oral microbiota is closely associated with the development and treatment of oral mucositis (Laborda-Illanes et al., 2020). Existing evidence indicates that microbiota modulation may help regulate the onset and severity of CIOM. However, current data remain insufficient to support its use as a monotherapy in clinical practice, and future studies are required to provide additional evidence regarding its safety profile and mechanistic pathways of action (Cereda et al., 2018).

## 6 Efficacy of probiotic in alleviating chemotherapy-induced oral mucositis

**Figure 2:**
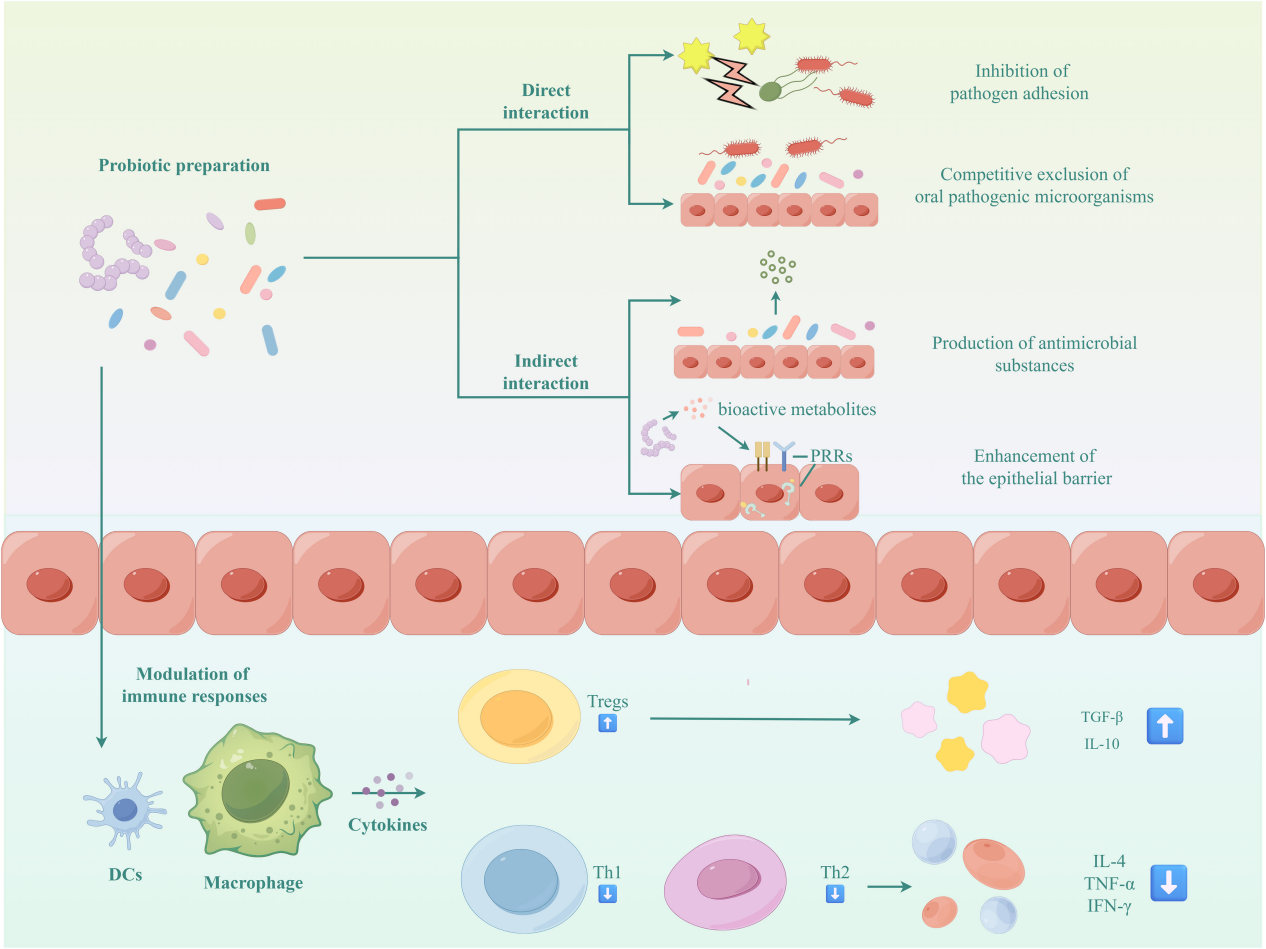
Ameliorative effects of probiotic preparations on chemotherapy-induced oral mucositis (CIOM). Through a multi-target synergistic mechanism of “bacteriostasis-repair-immunomodulation”, probiotic preparations mitigate chemotherapy-induced inflammation and damage to the oral mucosa. Probiotic preparations improve CIOM primarily via two interacting mechanisms: direct and indirect effects. Direct Action: Probiotics competitively exclude oral pathogenic microorganisms (by competing for colonization sites and nutrients) and produce antimicrobial substances (e.g., bacteriocins), directly inhibiting or reducing pathogenic bacterial loads to mitigate microbe-mediated mucosal damage at its source. Indirect Effects: Bioactive metabolites produced by probiotics (e.g., short-chain fatty acids) promote mucosal barrier repair and integrity by acting on pattern recognition receptors (PRRs) in epithelial cells. Additionally, probiotics and their metabolites modulate the function of immune cells such as macrophages and dendritic cells (DCs), thereby alleviating excessive inflammatory damage.

For a long time, the pathobiological mechanism of oral mucositis was thought to be limited to direct epithelial damage (Chikama et al., 2009). However, recent mechanistic studies have demonstrated that oral mucositis involves complex biological interactions throughout the entire mucosal tissue layer (epithelial cells, submucosal immune cells, extracellular matrix), encompassing multiple pathways including oxidative stress, inflammatory responses, and apoptosis. This updated understanding has identified additional potential therapeutic targets for OM (Shetty et al., 2022). Among these, probiotic preparations have emerged as a research focus due to their safety, efficacy, and cost-effectiveness (Sauruk da Silva et al., 2021). The mechanisms of action of probiotic preparations primarily encompass three aspects: ① Colonizing the oral mucosa to compete with pathogenic bacteria for adhesion sites and nutrients, thereby inhibiting their proliferation; ② Producing specific metabolites (e.g., SCFAs and bacteriocins) to maintain oral microecological homeostasis; ③ Regulating host immune responses by promoting the release of anti-inflammatory cytokines and suppressing inflammatory reactions, as shown in **Figure 2**.

A meta-analysis systematically summarizes the efficacy and tolerability of probiotic supplementation in preventing oral complications in cancer patients. The adjuvant use of probiotics can reduce the risk of oral mucositis during cancer treatment by 10% and the incidence of severe oral mucositis by 38% (Shafiei et al., 2025). A substantial body of published research evidence now demonstrates that probiotic preparations can modulate the oral microbiota by colonizing the oral mucosa, producing specific metabolites to maintain the ecological balance of the host microbiota, and achieving immune homeostasis (Han et al., 2020; Wang et al., 2021).The team led by Yan Wang applied Streptococcus salivarius in a mouse model of radiation-induced oral mucositis (RIOM). Results showed that this strain reduces nitrate reduction reactions in the oral cavity by downregulating the expression of the nitrate reductase gene (napA), thereby decreasing the abundance of Pasteurella and anaerobic bacteria. Concurrently, Streptococcus salivarius promotes the proliferation of oral commensals (e.g., Streptococcus spp.), thereby restoring oral microecological homeostasis (Wang et al., 2021). Han et al. observed accelerated healing of palatal lesions in mice treated with a mixture of Lactobacillus reuteri (probiotic) and *Porphyromonas gingivalis* (pathogen), compared with mice treated with the pathogen alone or the blank control group. This phenomenon may arise because reuterin produced by Lactobacillus reuteri degrades lipopolysaccharide (LPS) secreted by *Porphyromonas gingivalis*, thereby inhibiting LPS-induced NOD-like receptor family pyrin domain containing 3 (NLRP3) inflammasome activation (Han et al., 2020). Overall, probiotic preparations tailored for the human oral cavity demonstrate significant clinical potential. By regulating specific bacteria, they optimize the overall structure of the oral microbiota while maximally preserving the subject’s original microbiota, thereby avoiding adverse reactions associated with “microbiota replacement”. Compared to traditional treatments, probiotic preparations offer advantages of safety, controllability, proven efficacy, and low cost, making them promising interventions to alleviate treatment-related suffering and reduce costs for cancer patients. However, several critical challenges need to be addressed for the clinical translation of probiotic preparations, including strain-specific selection, optimization of delivery methods, and verification of long-term safety. Future efforts should focus on conducting more high-quality clinical studies to advance the standardized use of probiotic preparations in the clinical management of CIOM.

## 7 Conclusion and outlook

CIOM is a severe complication related to cancer treatment. Its occurrence and development are not caused by a single factor, but rather result from the complex interactions among the direct cytotoxicity of chemotherapy drugs, excessive activation of host immune-inflammatory responses, and dysbiosis of the oral microbiome. Among these, oral microbiota dysbiosis is not only the intersection where chemotherapy toxicity and immune responses influence each other, but also the core link driving the occurrence of CIOM and exacerbating its pathological process. Dysbiosis of the microbiota can lead to overproliferation of pathogenic bacteria, changes in metabolic products, and biofilm formed by the aggregation of pathogenic microorganisms. These factors directly exacerbate mucosal epithelial damage and continuously activate local immune-inflammatory cascades through pattern recognition receptors, thereby forming a vicious cycle.

Current clinical interventions primarily focus on symptomatic treatment, making it difficult to simultaneously achieve ideal efficacy, reliable safety, and low cost. Additionally, there is a severe lack of effective strategies for preventing CIOM before cancer treatment. Therefore, a deep understanding of the pathological mechanisms centered on microecological dysregulation is crucial for developing new prevention and treatment strategies. Probiotic preparations are becoming a research hotspot due to their potential in regulating oral microecological balance, inhibiting pathogen colonization, modulating local mucosal immunity, and alleviating inflammatory responses. They offer advantages of high safety and relatively low cost, which meet the urgent demand for accessibility in clinical translation.

However, we must also clearly recognize that current probiotic research still has limitations, primarily manifested in: a lack of targeted strain selection (blurred specificity), an unclear mechanism of action, and most crucially, issues with product standardization. This includes the absence of regulations for strain identification, preservation of viability, uniform dosage, and quality control, leading to difficulties in comparing and replicating research results, which hinders their widespread clinical application. Future research breakthroughs in this field should focus on the following directions: 1. Based on an in-depth understanding of the core pathological pathways of CIOM, screen or construct engineered probiotics that can specifically adhere, colonize, and target-regulate key pathogenic bacteria or immune nodes; 2. Develop ‘tailor-made’ probiotic combinations by integrating the individualized oral microbiome characteristics, genetic background, and chemotherapy regimens of cancer patients to achieve precise intervention. 3. By combining rigorous and standardized randomized controlled clinical trials, clarify its efficacy and safety in different tumor populations and at different stages of chemotherapy. Establish and promote clinical application standards for probiotic preparations, providing new clinical strategies for the prevention and treatment of CIOM.
